# Metabolic Syndrome Knowledge among Adults with Cardiometabolic Risk Factors: A Cross-Sectional Study

**DOI:** 10.3390/ijerph16010159

**Published:** 2019-01-08

**Authors:** Qun Wang, Sek Ying Chair, Eliza Mi-Ling Wong, Ruth E. Taylor-Piliae, Xi Chen Hui Qiu, Xiao Mei Li

**Affiliations:** 1 School of Nursing, Shenzhen University, 1066 Xue Yuan Road, Shenzhen 518055, China; qiuxichenhui@163.com; 2 The Nethersole School of Nursing, Faculty of Medicine, The Chinese Universityof Hong Kong, Shatin, Hong Kong; sychair@cuhk.edu.hk; 3 School of Nursing, The Hong Kong Polytechnic University, Hung Hom, Kowloon, Hong Kong; eliza.wong@polyu.edu.hk; 4 College of Nursing, University of Arizona, 1305 N. Martin, P.O. Box 210203, Tucson, AZ 85721, USA; rtaylor@nursing.arizona.edu; 5 Faculty of Nursing, College of Medicine, Xian Jiaotong University, 76 West Yanta Road, Xi’an 710061, China; roselee@mail.xjtu.edu.cn

**Keywords:** metabolic syndrome, knowledge, health education

## Abstract

Metabolic syndrome (MetS) is a cluster of cardiometabolic risk factors. Many people may be unaware of their risk for MetS. A cross-sectional, descriptive study was conducted among hospitalized patients with at least one cardiometabolic risk factor in Mainland China. This study assessed the MetS knowledgelevel(through MetS Knowledge Scale, MSKS) and examined the potential predictors by regression analysis. A total of 204 patients aged 58.5 ± 10.1 years (55% males) participated in this study. The majority of participants had no history of hypertension (54%), dyslipidemia (79%), or diabetes (85%). However, 56% of these participants had at least three cardiometabolic risk factors, indicating the presence of MetS. The average MSKS was very low (mean = 36.7 ± 18.8, possible range = 0–100), indicating the urgent needs of MetS education in current practice. Predictors of better MetS knowledge included higher educational level, history of dyslipidemia, and normal high-density lipoprotein cholesterol (F (8, 195) = 9.39, adjusted R^2^ = 0.192, *p* < 0.001). In conclusion, adults with cardiometabolic risk factors are at risk of developing MetS, but with a low level of knowledge. Specific health education on MetS should be provided, particularly for those with limited formal education or inadequate lipid management.

## 1. Introduction

Metabolic syndrome (MetS) is a cluster of cardiometabolic risk factors, including central obesity, elevated blood pressure (BP), hyperglycemia, elevated triglyceride (TG), and low high-density lipoprotein cholesterol (HDL-C) [1]. Consistent with the epidemic of overweight and inactive lifestyles, the prevalence of MetS has been steadily increasing worldwide [2]. The prevalence of MetS is 28.5% to 38.5% in western populations [3,4,5] and 24.1% in the Chinese population [6]. Although the prevalence was lower than that in western countries, China has the greatest number of MetS population in the world. MetS has close relationship with type 2 diabetes mellitus (DM) and cardiovascular diseases (CVD). Those with MetS had a relative risk of 2.99–6.08 for developing DM, a two-fold increased risk in developing CVD, and 1.5-fold increase in all cause mortality [7,8]. Given the increasing prevalence, MetS inflicts great challenges, along with heavy medical burdens to individuals and the healthcare system [9].

Lifestyle modifications, such as regular exercise, healthy diet, and weight control, are recommended as first-line interventions for MetS management [2,10]. All of these lifestyle modifications involve behavioral changes. Adequate knowledge of the prevention and management of MetS would facilitate people’s adaptation of healthy behaviors [11]. Hospitals are expected to take the roles of providing MetS education for the patients, especially those already with cardiometabolic risk factors. Understanding patients’knowledge of MetSwill not only indicate the health education services in clinical practice and provide improvement suggestions. More importantly, the findings would aid healthcare professionals to identify what educational efforts are needed. 

Several studies have assessed MetS awareness or CVD-related knowledge [12,13,14]. Only 12.5% to 29.0% of the participants had heard about MetS [13,14], and fewer participants (10.0%) understood the definition of MetS [12]. Only a few studies have investigated the knowledge specifically on MetS [15,16,17,18,19,20,21]. These studies used either a single item or self-developed questionnaires to assess the level of MetS knowledge among general population or healthcare providers [15,16,17,18,19,20]. The items like “have you ever heard about MetS” could not provide comprehensive and reliable assessment of respondents’ knowledge. One study in Hong Kong applied a validated MetS knowledge scale and reporteda poor understanding of MetSamong community residents [21]. That study excluded elderly people (≥65 years) or those with knownCVD. The lipid variables (TG and HDL-C) were not measured either. Given the unique socio-cultural characteristics and special healthcare system in Hong Kong, their study findings could neither indicate the level of MetS knowledge among adults in Mainland China nor imply current MetS education services in clinical practice. The aim of the current study was to assess knowledge of MetS and to explorethe potential predictors among adults with cardiometabolic risk factors in Mainland China. The study findings would provide valuable information for developing effective MetS management strategies in this at-risk population.

## 2. Materials and Methods

### 2.1. Ethics Statement

The study was approved by the Survey and Behavioral Research Ethics Committee of the Chinese University of Hong Kong (No. SBRE-20121202). The study conformed to the Declaration of Helsinki guidelines. A letter explaining the purpose and details of the study was provided to each potential participant. Written consent was obtained from each participant.

### 2.2. Design and Settings

This was a cross-sectional, descriptive study. The study was conducted in two university-affiliated hospitals in the city of Xian, Mainland China. Both study hospitals are public general hospitals with over 1000 beds and they have obtained the III-A-level certification in China, indicating the top-level of hospital with strong abilities in medical service, education, and research. Both medicine and surgical departments of the hospital were involved in the study.

### 2.3. Study Participants

The study participants were Chinese adults with at least one cardiometabolic risk factor. In the current study, cardiometabolic risk factors were defined as the five risk factors following the MetS definition [1]: (1) central obesity: waist circumference (WC) ≥ 90 cm for males, or ≥80 cm for females (specific criteria for Asian population); (2) elevated BP: ≥130/85 mmHg; or taking antihypertensive medications; (3) hyperglycemia: fasting plasma glucose (FPG) ≥ 5.6 mmol/L (100 mg/dL), or taking medications; (4) elevated TG: ≥1.70 mmol/L (150 mg/dL), or taking medications; and, (5) low HDL-C: <1.03 mmol/L (40 mg/dL) in males, or <1.29 mmol/L (50 mg/dL)in females; or, taking medications. People with three or more factors are defined as having MetS [1].

Study inclusion criteria were adults aged 18 years and older, with one or morecardiometabolic risk factor, as mentioned above, and medically stable. Those could not be able to communicate in Mandarin or with impaired bilateral hearing were excluded. 

The study sample size was estimated based on the power analysis for multiple regression. A minimum of 194 participants provided 80% power to achieve a small to medium effect size (*r* = 0.2) at a 5% level of significance [22].

### 2.4. Data Collection

The study employed a convenient sampling approach. The researcher firstly screened the eligibility of patients in the study hospitals by reviewing the medical records. Potential participants that met the study criteria were invited and given a letter introducing the purpose and details of the study. After obtaining the written consent, the researcher started the data collection procedure. To maintain consistency, all questionnaires were administered by the same researcher through face-to-face interviews. Each interview lasted 10 min to 15 min.

### 2.5. Measurements

#### 2.5.1. Self-Reported Demographic and Clinical Characteristics

A structured data collection questionnaire was used to obtain the demographics and clinical characteristics of the participants. Demographic characteristics included age, gender, marital status, educational level, and occupation. Medical history of hypertension, dyslipidemia and diabetes, and anthropometric data of BMI and BP were obtained from the medical records. The study hospital did not measure WC in the routine practice. WC was measured by the researcher using a tape at the midpoint of the lower border of the ribs and the iliac crest in a horizontal plane. The results of FPG, TG, and HDL-C were retrieved from the latest laboratory test records during the current hospitalization.

#### 2.5.2. MetS Knowledge Scale

The 10-item MetS Knowledge Scale (MSKS) was used to assess knowledge of MetS [14]. The MSKS contains three subscales: definition and diagnosis of MetS (five items), complications of MetS (two items), and prevention of MetS (three items). Each item has five choices, with one correct answer and one choice of “do not know”. For example, the seconditem “What is the correct threshold of WC for central obesity in males?” has the choices of “A. ≥80 cm; B. ≥90 cm; C. ≥100 cm; D. ≥110 cm; E. Do not know.” Each item is scored as 10 (the correct answer) or 0 (incorrect answer or ‘do not know’). The total score is the sum of each item score, with a possible range from 0 to 100. A higher score indicates a better understanding of MetS knowledge. The MSKS had been validated among Chinese and MetSpopulations, with a good content validity (CVI = 98.1%) and internal consistency (Cranach’s α = 0.69–0.77) [14,21]. Internal consistency of the MSKS in our study was good (Cranach’s α = 0.79).

### 2.6. Statistical Analyses

Characteristics of the participants were described as mean ± standard deviation (SD), median (inter-quartile range, IQR), or frequency (percentage), as appropriate. Bivariate analyses using independent t-tests, one-way analysis of variation (ANOVA), or Pearson correlation coefficients were conducted to compare the MetS knowledge among participants with different characteristics. Multiple linear regression with the backward elimination method was conducted to explore potential predictors of MetS knowledge. The total score of MSKS was analyzed as the dependent variable. To reduce the possibility of excluding important factors from the regression model, factors with a *p*-value less than 0.20 in bivariate analyses were identified as independent variables [23]. All the tests were two-tailed and the significance level was set at *p* < 0.05. SPSS version 20.0 was used for statistical analyses.

## 3. Results

### 3.1. Characteristics of the Participants

A total of 252 patients were invited, and 204 agreed to participate, with a response rate of 80.95%. The study sample included 113 (55.4%) males aged 35 to 79 years (mean = 58.5, SD = 10.1). Most participants (96.1%) were married, with less than 12 years formal education (77%). A small part of participants reported history of dyslipidemia (21.1%) or DM (14.7%), and 46.1% of them had hypertension. Most participants (62.3%) were overweight or obese. Low HDL-C (68.6%) was the most common factor, followed by central obesity (65.7%), elevated BP (62.7%), and hyperglycemia (50.5%). More than half (55.9%) of the participants had at least three cardiometabolic risk factors, indicating the presence of MetS (Table 1).

The continuous data of participants’ cardiometabolic characteristics arepresented in Table 2. Consistent with the categorical results (Table 1), the mean BMI (24.18 Kg/m^2^) and WC (males: 91.24 cm, females: 85.38 cm) indicated the high prevalence of over-weight and central obesity among the participants. Their mean BP levels (systolic: 130.73 mmHg; diastolic: 81.22 mmHg) were higher than the MetS criteria of 130/80 mmHg. Although the median FPG (5.44 mmol/L) and TG (1.41 mmol/L) were within normal range, the median HDL-C levels (males: 0.93 mmol/L; females: 1.10 mmol/L) were lower than the criteria of MetS definition.

### 3.2. MetS Knowledge of the Participants

Table 3 presents the results of MSKS on total scale, subscales, and individual items. The mean total score of MSKS was very low (mean = 36.7, SD = 18.8, possible range: 0 to 100). On average, each participant provided 3.6 correct answers for the whole scale, and every participant answered “do not know” for 4.5 items.

The subscale of definition and diagnosis had the lowest mean score (5.24, range: 0 to 50) and the lowest rate of correct answers (10.5%), followed by the subscale of complications (9.17, range: 0 to 20; correct answer rate: 45.8%). The highest score was reported on Mets prevention (mean = 22.3, range: 0 to 30) with 74.2% correct answers.

For individual items of MSKS, the item on medical management received the most correct answers (84.3%), followed by the item on pertaining to self-care (72.1%). Items on thresholds of hyperglycemia, elevated BP, and WC in females had the least correct answers (3.4%, 9.3%, and 10.3%, respectively). The most “do not know” answers were received by the secondand thirditems on thresholds of WC in males (73.5%) and females (67.2%).

### 3.3. Predictors of MetSKnowledge

No significant correlation was detected between age and MSKS score (Pearson correlation *r* = 0.081, *p* = 0.249). Bivariate analyses revealed that participants with different occupations (*p* = 0.003), educational levels (*p* < 0.001), history of dyslipidemia (*p* = 0.001), and HDL-C status (*p* = 0.028) had different MSKS scores (Table 1).

In the regression analysis, the total score of MSKS was analyzed as the dependent variable. Seven factors with *p*-values of less than 0.20 in bivariate analyses were analyzed as independent variables in the regression analysis [23], including occupation, educational level, and history of dyslipidemia, presence of central obesity, elevated BP and low HDL-C, and presence of MetS (Table 1). In the final regression model (Table 4), history of dyslipidemia (*p* = 0.003), 9 to 12 years of formal education (*p* = 0.001), greater than 12 years of formal education (*p* < 0.001), and low HDL-C levels (*p* = 0.027) were significant predictors of MetS knowledge. These factors explained 19.2% of variance in MetS knowledge (*p* < 0.001), indicating a medium to large effect size [22].

## 4. Discussion

This was the first study to examine the knowledge of MetS and its predictors, using a validated instrument, among adults with cardiometabolic risk factors in Mainland China. In the current study, the participants were at high risk of developing MetS, among which more than half had MetS. The study was conducted in the best hospitals in Mainland China, where physicians and nurses were expected to provide health education related to patients’ cardiometabolic risk factors. However, patients revealed poor knowledge about MetS, indicating the urgent needs of MetS education in current practice. Education level and lipid management were significant predictors of MetS knowledge, indicating the need to develop educational strategies for this at-risk population.

The mean MSKS score of 36.7, out of the 100 total score. About two-thirds of the participants (67.2%) obtained a MSKS score of less than 50. The poor knowledge level was similar toprevious studies [15,16,17,18,19,20]. The Hong Kong study reported a higher MSKS score in community residents (mean = 44.9), which may be ascribed to the public campaigns on MetS [21], indicating the importance of MetS education in public. Before the hospitalization, our participants also lived in communities. Their poor understanding of MetS implied the lack of MetSeducation for the public populations.

In the current study, more than halfof the participants had MetS. However, they could only correctly answer 10.5% of the items onMetS definition and diagnosis. Without an adequate understanding of the definition and diagnosis, patients could not be aware of the presence of MetS, or their increased risks for developing DM or CVD. Similarly, a prior European study reported that 42.2% of the patients with early DM and/or MetS thought that they had good or excellent health, though 39.6% were unaware of their CVD risk [24]. Only 18.1% of our participants correctly answered the item on MetS definition. Consistently, 12.5% of the respondents in United States understood the definition of MetS [12]; previous surveys in China and Hungary reported that 10% and 29% of their respondents had heard about MetS, respectively [13,25]. These findings implied the urgent need of MetS-specific health education all over the world.

The current participants’ poor understanding about MetS diagnosis was evident and significant. Firstly, few participants (10.3–11.3%) knew the criteria for central obesity (the secondand thirditem in MSKS). Despite the critical role of central obesity in developing CVD and DM [1,2], the study hospitals did not include WC measurement in routine assessment. These findings indicated the ignorance of central obesity in current clinical practice. Secondly, few participants knew the threshold for elevated BP (9.3%) or FPG (3.4%). Notably, 107 (52.5%) participants regarded ≥ 140/90 mmHg as elevated BP and 47 (23.0%) selected FPG ≥ 6.1 mmol/L as hyperglycemia. They confused the criteria of MetS with that for hypertension and DM during the study period. The Hong Kong study also reported low scores in these items on MetS diagnosis [21]. These findings may, in part, be explained by the greater emphasis on disease diagnoses rather than on disease prevention in current health education. With poor knowledge of the normal ranges for WC, BP, or FPG, these patients would not realize the urgency of implementing disease management. Thus, specific health education targeting MetS and early disease prevention should be enhanced in the healthcare system.

The subscale of prevention and complications received a bit more correct answers (74.2% and 45.8%, respectively). When compared with the study in Hong Kong [21], the current participants revealed a higher mean score in MetSprevention (22.3 vs. 16.9). The educational emphasis on healthy lifestyles in hospitals may explain the higher scores in MetS prevention. The imbalanced knowledge of MetS across these subscales is similar to the findings on people’s knowledge structure in a German study [26]. The German study illustrated that people usually knew more about the enabling conditions and treatments of diseases, but lacked knowledge of disease mechanisms [26].

In the current study, participants with different demographic and clinical characteristics revealed significantly different knowledge levels, indicating the importance of assessing people’s educational needs and designing tailor-made interventions. Consistent with previous findings [18,21,27,28], people with higher education in this study were more knowledgeable about MetS. Higher education is associated with better learning abilities and more effective communication with healthcare providers [29,30,31]. Specific strategies for targeting health education among persons with lower literacy are needed, such as using simpler language or interactive educational methods [32]. The bivariate analyses revealed similar findingstoprevious studies that people with administrative and clerical occupations had more MetS knowledge [11,21,33,34]. However, occupation was not a significant predictor of MetS knowledge in the final regression model, which may be caused by the dominant influences of education.

In prior published studies, age was significantly correlated with MetS knowledge [17,18,19,21]. However in our study among the at-risk population, age was not associated with MetS knowledge. Although patients with MetS reported a higher mean MSKS score than those without MetS (39.0 vs. 31.8), no significant statistical difference was detected (*p* = 0.116). Moreover, the presence of MetS was not included in the final regression model, indicating the non-significant influence of these combined risk factors. Instead, participants with a history of dyslipidemia or normal HDL-C level had more MetS knowledge. These participants may have received MetS-related education after their dyslipidemia diagnosis. Furthermore, once the participants were aware of the dyslipidemia diagnosis, they may pay attention to the self-learning of related knowledge. People with a normal HDL-C level also knew more about MetS, indicating the beneficial effects of knowledge on disease management. The actual cause-effect relationship between MetS knowledge and lipid management needs to be examined in future studies.

There are some limitations in the study. Firstly, this study used convenience sampling to recruit participants from the inpatient departments of two hospitals. Attentions should be paid when generalizing the findings to other populations. Future studies could measure the MetS knowledge among various populations in diverse settings (e.g., communityresidents, students, and working populations). Secondly, only 19% of the variance in MetS knowledge could be explained by the studied factors. Future studies may examine the cultural or other potential predictors of MetS knowledge, so that specific interventions can be indicated. The data collection process also indicated that the 10-item MSKS is a useful instrument to provide quick measurement of patients’ MetS knowledge. This instrument could be applied by healthcare professionals in clinical settings.

## 5. Conclusions

Understanding patients’ MetS knowledge and predictors is essential for healthcare professionals to identify current MetS education demands and to facilitate the development of effective interventions for MetS prevention and management. Adults with cardiometabolic risk factors are at risk of developing MetS, but had poor knowledge about MetS. The educational needs of this at-risk population, particularly those with limited formal education or inadequate lipid management, along with targeted health education strategies, needs to be systematically implemented in clinical practice.

## Figures and Tables

**Table 1:** Participants’ Characteristics and Knowledge of Metabolic Syndrome (n = 204).

Characteristics	*n* (%)	MetS Knowledge (Mean ± SD)	*t*-Test or ANOVA ^†^*p*-Value
**Age**			0.472 ^†^
35–49 years	38 (18.6)	36.3 ± 18.9	
50–59 years	71 (34.8)	34.1 ± 17.9	
60–69 years	65 (31.9)	38.5 ± 18.9	
70–79 years	30 (14.7)	39.3 ± 20.8	
**Gender**			0.214
Male	113 (55.4)	38.1 ± 18.3	
Female	91 (44.6)	34.8 ± 19.4	
**Marital status**			0.203
Married	196 (96.1)	36.3 ± 19.0	
Widowed	8 (3.9)	45.0 ± 14.1	
**Occupation ^†^**			0.003 **
Unemployed	8 (3.9)	23.8 ± 24.4	
Retired	79 (38.7)	39.1 ± 19.5	
Farmer	59 (28.9)	30.7 ± 16.1	
Industrial worker	20 (9.8)	37.5 ± 19.4	
Administrative/clerical work	38 (18.6)	43.2 ± 16.9	
**Education level ^†^**			<0.001 ***
≤6 years	36 (17.7)	26.9 ± 17.0	
7 to 9 years	60 (29.4)	31.3 ± 17.6	
9 to 12 years	62 (30.4)	40.6 ± 19.5	
>12 years	46 (22.5)	45.9 ± 15.3	
**History of hypertension**			0.488
Yes	94 (46.1)	37.7 ± 18.4	
No	110 (53.9)	35.8 ± 19.2	
**History of dyslipidemia**			0.001 **
Yes	43 (21.1)	45.1 ± 16.5	
No	161 (78.9)	34.4 ± 18.8	
**History of diabetes**			
Yes	30 (14.7)	38.0 ± 16.1	0.676
No	174 (85.3)	36.4 ± 19.3	
**Body Mass Index (Kg/m^2^)**			0.747
Normal (≤23.0)	77(37.7)	37.9 ± 20.5	
Overweight (23.0–25.0)	44 (21.6)	36.4 ± 16.8	
Obese (≥25.0)	83 (40.7)	35.7 ± 18.4	
**MetS risk factors**			
**Central Obesity** (WC: males ≥ 90 cm or females ≥ 80 cm)			0.176
Yes	134 (65.7)	35.4 ± 19.0	
No	70 (34.3)	39.1 ± 18.5	
**Elevated BP** (BP ≥ 130/85 mmHg)			0.168
Yes	128 (62.7)	38.0 ± 18.8	
No	76 (37.3)	34.1 ± 18.8	
**Hyperglycemia** (FPG ≥ 5.6 mmol/L)			0.245
Yes	103 (50.5)	35.1 ± 17.8	
No	101 (49.5)	38.2 ± 19.9	
**Elevated TG** (TG ≥ 1.7 mmol/L)			0.939
Yes	75 (36.8)	36.8 ± 18.6	
No	129 (63.2)	36.6 ± 19.0	
**Low HDL-C** (males: <1.29 mmol/L or females: <1.03 mmol/L)			0.028 *
Yes	140 (68.6)	34.7 ± 18.3	
No	64 (31.4)	40.9 ± 19.4	
**Number of MetS risk factors ^†^**			0.386
1	29 (14.2)	41.7 ± 21.0	
2	61 (29.9)	37.7 ± 18.4	
3	43 (21.1)	32.8 ± 18.8	
4	49 (24.0)	36.1 ± 17.8	
5	22 (10.8)	35.9 ± 19.4	
**Presence of MetS**			0.116
Yes	114 (55.9)	31.8 ± 18.4	
No	90 (44.1)	39.0 ± 19.2	

The categorical data of participants’ characteristics.SD: standard deviation. BP = blood pressure; FPG: Fasting plasma glucose; HDL-C: high-density lipoprotein cholesterol; MetS: metabolic syndrome; TG: triglycerides; WC: waist circumference; ^†^ ANOVA: analysis of variance. * *p* < 0.05; ** *p* < 0.01; *** *p* < 0.001.

**Table 2:** Participants‘Cardiometabolic Characteristics (n = 204).

Cardiometabolic Characteristics	Mean/Median	Standard Deviation/Inter-Quartile Range
**BMI (Kg/m^2^)**	24.18	3.28
**Waist circumference (cm)**		
Males	91.24	10.15
Females	85.38	10.56
**Blood pressure (mmHg)**		
Systolic	130.73	19.04
Diastolic	81.22	12.60
**FPG (mmol/L)** ^†^	5.44	(4.70, 6.69)
**Triglyceride(mmol/L)** ^†^	1.41	(1.04, 1.93)
**HDL-C (mmol/L)** ^†^		
Males	0.93	(0.84, 1.09)
Females	1.10	(0.92, 1.33)

^†^ Skewed distributed variables presented as mean (inter-quartile range); BMI: Body mass index; FPG: Fasting plasma glucose; HDL-C: high-density lipoprotein cholesterol; LDL-C: low-density lipoprotein cholesterol.

**Table 3:** Metabolic Syndrome Knowledge Scale Scores (n = 204).

Subscales and Items	Score Mean ± SD	Correct Answer *n* (%)	Do Not Know *n* (%)
**Subscale: Definition/Diagnosis of MetS (range 0–50)**	*5.24 ± 7.72*	*107 (10.5)*	*607 (59.5)*
1. Defines MetS	1.81 ± 3.86	37 (18.1)	134 (65.7)
2. Threshold of WC for males	1.13 ± 3.17	23 (11.3)	150 (73.5)
3. Threshold of WC in females	1.03 ± 3.05	21 (10.3)	137 (67.2)
4. Threshold of elevated BP	0.93 ± 2.91	19 (9.3)	68 (33.3)
5. Threshold of hyperglycemia	0.34 ± 1.82	7 (3.4)	118 (57.8)
**Subscale: Complications of MetS (range 0–20)**	*9.17 ± 8.17*	*187 (45.8)*	*195 (47.8)*
6. MetS complications	4.90 ± 5.01	100 (49.0)	99 (48.5)
7. Effects of high WC	4.26 ± 4.96	87 (42.6)	96 (47.1)
**Subscale: Prevention of MetS (range 0–30)**	*22.3 ± 9.51*	*454 (74.2)*	*116 (18.9)*
8. Factors associated with MetS	6.62 ± 4.74	135 (66.2)	45 (22.1)
9. Self-care for persons with MetS	7.21 ± 4.50	147 (72.1)	43 (21.1)
10. Medical management for MetS	8.43 ± 3.64	172 (84.3)	28 (13.7)
**Total scale score (range 0–100)**	36.7 ± 18.8	748 (36.7)	918 (45.0)

BP: blood pressure; MetS: metabolic syndrome; WC: waist circumference. SD: standard deviation.

**Table 4:** Predictors of Metabolic Syndrome Knowledge (n = 204).

Variables	Coefficient	Standardized Coefficient	95% CI for Coefficient		*p*-Value
History of dyslipidemia	0.911	0.198	0.320	1.501	0.003
9 to12-year education ^†^	1.207	0.295	0.492	1.922	0.001 ^†^
>12-year education ^†^	1.637	0.364	0.869	2.406	<0.001 ^†^
Low HDL-C	−0.592	−0.146	−1.116	−0.067	0.027

F_(8,195)_ = 9.393, adjusted R^2^ = 0.192, *p* < 0.001. HDL-C: high-density lipoprotein cholesterol; ^†^ education ≤ 6 years as the reference group.
